# A specific form of prefibrillar aggregates that functions as a precursor of amyloid nucleation

**DOI:** 10.1038/s41598-017-18390-y

**Published:** 2018-01-08

**Authors:** Naoki Yamamoto, Shoko Tsuhara, Atsuo Tamura, Eri Chatani

**Affiliations:** Graduate School of Science, Kobe University, 1-1 Rokkodai-cho, Nada-ku, Kobe, Hyogo 657-8501 Japan

## Abstract

Non-fibrillar protein aggregates that appear in the earlier stages of amyloid fibril formation are sometimes considered to play a key role in amyloid nucleation; however, the structural features of these aggregates currently remain unclear. We herein identified a characteristic pathway of fibril formation by human insulin B chain, in which two major species of prefibrillar aggregates were identified. Based on the time-resolved tracking of this pathway with far-UV circular dichroism (CD) spectroscopy, dynamic light scattering (DLS), and ^1^H-NMR spectroscopy, the first prefibrillar aggregate with a hydrodynamic diameter of approximately 70 nm accumulated concomitantly with the formation of a β-sheet structure, and the size further evolved to 130 nm with an additional structural development. These prefibrillar aggregates were metastable and survived at least 24 hours as long as they were maintained under quiescent conditions. The energy barrier for nucleation was overcome by shaking or even by applying a single short ultrasonic pulse. Furthermore, an investigation where nucleation efficiency was monitored by fibrillation rates with varying the timing of the ultrasonic-pulse treatment revealed that the second prefibrillar aggregate specifically produced amyloid nuclei. These results suggest that the second form of the prefibrillar aggregates acts as a direct precursor for the amyloid nucleation.

## Introduction

Protein misfolding sometimes results in protein aggregation. Amyloid fibrils are a representative class of these aggregates, which are now known to be related to serious diseases such as Alzheimer’s and Parkinson’s diseases as well as type 2 diabetes^1,2^. Amyloid fibrils possess a common architecture composed of β-sheet structures that align in the fibril axis by hydrogen bonds^3^, although an exception has very recently been reported^4^. Fibril formation shares similarities with crystallization because of the repetitive construction of amyloid structures. A nucleation-dependent mechanism, in which nuclei for fibril formation are formed and free monomers or oligomeric species then bind to nuclei leading to the elongation of amyloid fibrils, has been proposed as a fundamental scheme. Nucleation typically functions as a rate-limiting step for the production of amyloid fibrils, and, thus, it is important to elucidate the molecular mechanisms underlying amyloid nucleation for clarification of the etiopathogenesis of diseases associated with the formation of amyloid fibrils.

One of the simplest and most classical schemes for amyloid nucleation is the appearance of nuclei in one step without any thermostable intermediates. However, early presence of non-fibrillar aggregates is frequently observed, which have recently been focused on as an intermediate species involved in the nucleation process or structural entities responsible for cytotoxicity. A large variety of protein oligomers, protofibrils, and annular, cylindrical, and other types of aggregates have been identified in recent years^5–7^. Some aggregates have been shown to function as species that form amyloid fibrils through structural reconstruction^8–12^ while some other aggregates never contribute to amyloid formation^13,14^. It is thus important to distinguish the on-pathway intermediates responsible for fibril formation based on their specific properties; however, the aggregates reported to date have varied in size, morphology, and structure, and difficulties are associated with identifying the properties influencing the conversion to nuclei. Therefore, prefibrillar aggregates that are transiently formed prior to amyloid fibril formation need to be trapped and characterized to obtain a more unified understanding of the formation of prefibrillar aggregates occurring prior to amyloid fibril formation.

**Figure 1:**
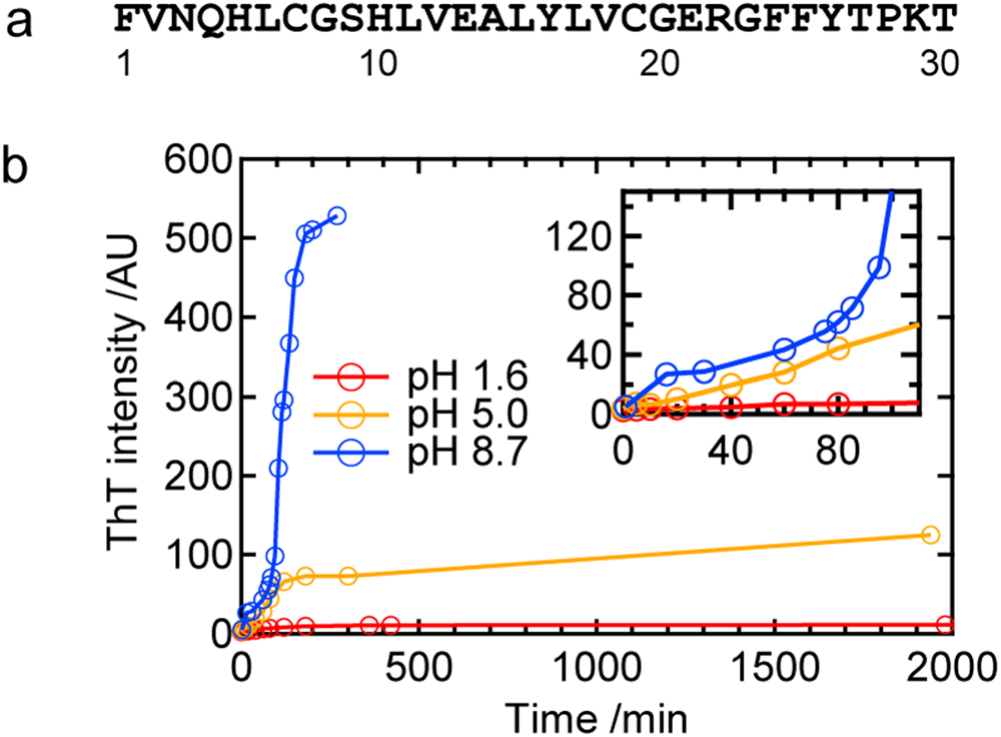
Fundamental properties of amyloid fibril formation by the insulin-derived B chain peptide. (**a**) The amino acid sequence of the B chain. (**b**) Time course of ThT fluorescence intensities at pH 1.6, 5.0, and 8.7. The inset shows the time course in the early stage.

In the present study, we examined the fibrillation reaction of an insulin-derived peptide, the B chain (Fig. 1a), with a focus on prefibrillar aggregates that may be generated in this process. Insulin is one of the most important hormone proteins produced by the β cells of pancreatic islets and regulates blood glucose levels. It is regarded as a good model for the study of amyloid fibril formation because of its strong propensity to form amyloid fibrils *in vitro* ^15,16^. Insulin is composed of A and B chains, which are covalently connected to each other through two disulfide bonds, and these two polypeptide chains independently form amyloid fibrils even after they are separated by the cleavage of disulfide bonds^17–19^. The B chain is more hydrophobic than the A chain and, thus, precipitates particularly around the isoelectric point due to its low solubility^18^. These findings suggest that this peptide has the potential to become a good model for studying the nucleation mechanism due to its propensity to form prefibrillar aggregates that appear prior to the maturation of the amyloid fibrils.

We herein demonstrated that the insulin B chain formed prefibrillar aggregates under slightly basic conditions. By performing time-resolved tracking of its aggregation process, we clarified that at least two types of prefibrillar intermediates that differ in size and secondary structure sequentially appeared. Furthermore, we found that the prefibrillar intermediate states were kept stable as long as they were not stimulated by any external mechanical forces. On the other hand, once agitation, such as shaking or a short ultrasonic pulse, was applied, prefibrillar intermediates were promptly converted to amyloid fibrils. Based on the results obtained from detailed investigations of nucleation after ultrasonic pulse triggering, a specific form of prefibrillar aggregates appears to contribute to the induction of amyloid nucleation.

## Results

### Detection and structural characterization of prefibrillar aggregates

To seek conditions under which amyloid fibrils of the B chain grow via intermediates, we initially traced time-dependent changes in thioflavin T (ThT) fluorescence intensities at several pH values. This analysis was performed under agitated conditions, i.e., sample liquids were shaken constantly. As a result, we successfully observed gradual time-dependent evolutions of ThT fluorescence emission at pH 1.6, 5.0, or 8.7 (Fig. 1b). Especially at pH 8.7, a stepwise increase in the ThT intensity was observed, at which an initial increase slowed at approximately 80 min and another increase started and intensity again reached a plateau at approximately 200 min (Fig. 1b, blue plot). Although the timing of the later increase of ThT fluorescence intensity and its final value tended to vary among experiments, the two-phase increase in ThT fluorescence intensity was reproducible (Fig. S1a). We thus focused on the pH value of 8.7 to identify and characterize prefibrillar intermediates possibly formed in the process of fibrillation.

**Figure 2:**
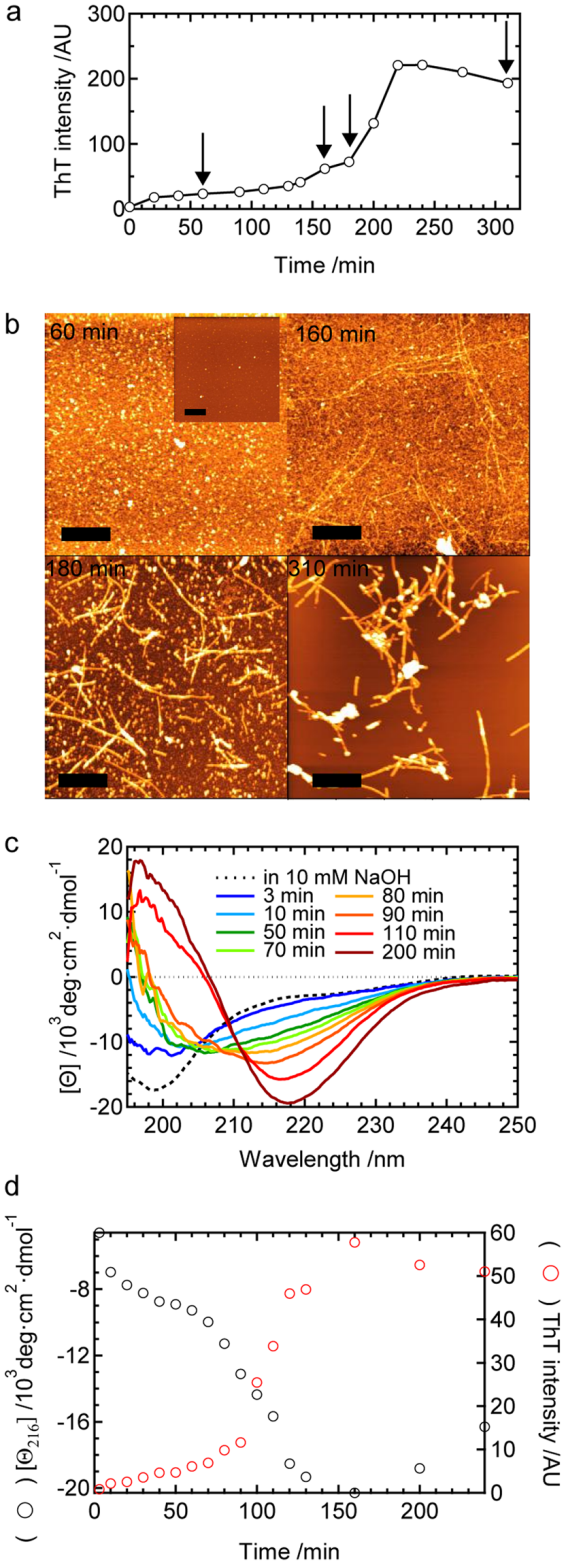
Time course of amyloid fibril formation by the B chain at pH 8.7 under agitated conditions. (**a**) Time course of ThT fluorescence intensity and (**b**) corresponding time dependence of aggregation, as monitored by AFM images. In panel a, time points of AFM measurements are indicated by arrows. The scale bars in panel b represent 1 μm. The inset shows an AFM image taken at time zero (immediately after the reaction started). (**c**) Time-dependent changes in CD spectra. The spectrum obtained in 10 mM NaOH is also shown by a dashed line as a reference. (**d**) Time dependence of molar ellipticity at 216 nm [*θ*_216_] during amyloid fibril formation. Corresponding time course of ThT fluorescence intensity is also shown.

Figure 2a,b show the time course of ThT fluorescence intensity and corresponding atomic force microscopy (AFM) images, respectively. In this analysis, ThT time course was monitored simultaneously with the AFM sampling using the same sample liquid. Soon after the first ThT fluorescence increase became saturated, granular particles with sizes ranging between 3 and 5 nm were observed (Fig. 2b, 60 min), which suggested the accumulation of prefibrillar aggregates. We confirmed that the number of particles was much fewer at time zero (Fig. 2b, inset). After the completion of the 1st phase of the ThT fluorescence increase, fibril structures began to appear co-existent with the particles and the relative amount of fibrils increased with time (Fig. 2b, 160 min and 180 min). Fibrils then became dominant when ThT fluorescent intensity reached plateau (Fig. 2b, 310 min).

In order to characterize the transient formation of prefibrillar aggregates, the time-resolved evolution of the secondary structures of the B chain was also monitored by circular dichroism (CD) spectra. During the process of the formation of prefibrillar aggregates and even in the fibril formation process, the sample was well dispersed only with a slight degree of turbidity. Thus reliable CD measurements were performed down to 195 nm without any correction for sample precipitation under the present experimental conditions. In the monomeric state of the B chain before aggregation (in 10 mM NaOH), the spectrum possessed a minimum at approximately 200 nm, which is typical for a random-coil structure (Fig. 2c, dotted line)^20^. The shape of the spectrum changed immediately after the reaction started, thereby confirming the formation of prefibrillar aggregates. The spectral value at approximately 220 nm decreased, while that of 200 nm increased with an isoellipticity point at approximately 204 nm up to 70 min (Fig. 2c, 10–70 min), suggesting that prefibrillar aggregates included some organized secondary structures. At 80 min and thereafter, the spectrum started to change toward a typical spectrum for the β-sheet structure, which possessed a minimum at approximately 216 nm (Fig. 2c, 80–200 min). When the values of molar ellipticity at 216 nm were plotted against reaction time, biphasic structural changes were revealed, which were in conjugation with the time course of of ThT fluorescence intensity at 485 nm obtained by using the same sample liquid as that of the CD measurement (Fig. 2d). These ThT, AFM, and CD results demonstrated that granular particles defined as prefibrillar aggregates accumulate with secondary structural formation in the early phase, followed by the fibril formation in the later phase.

### Time-resolved evolution of the structure of prefibrillar intermediates tracked under non-agitated conditions

**Figure 3:**
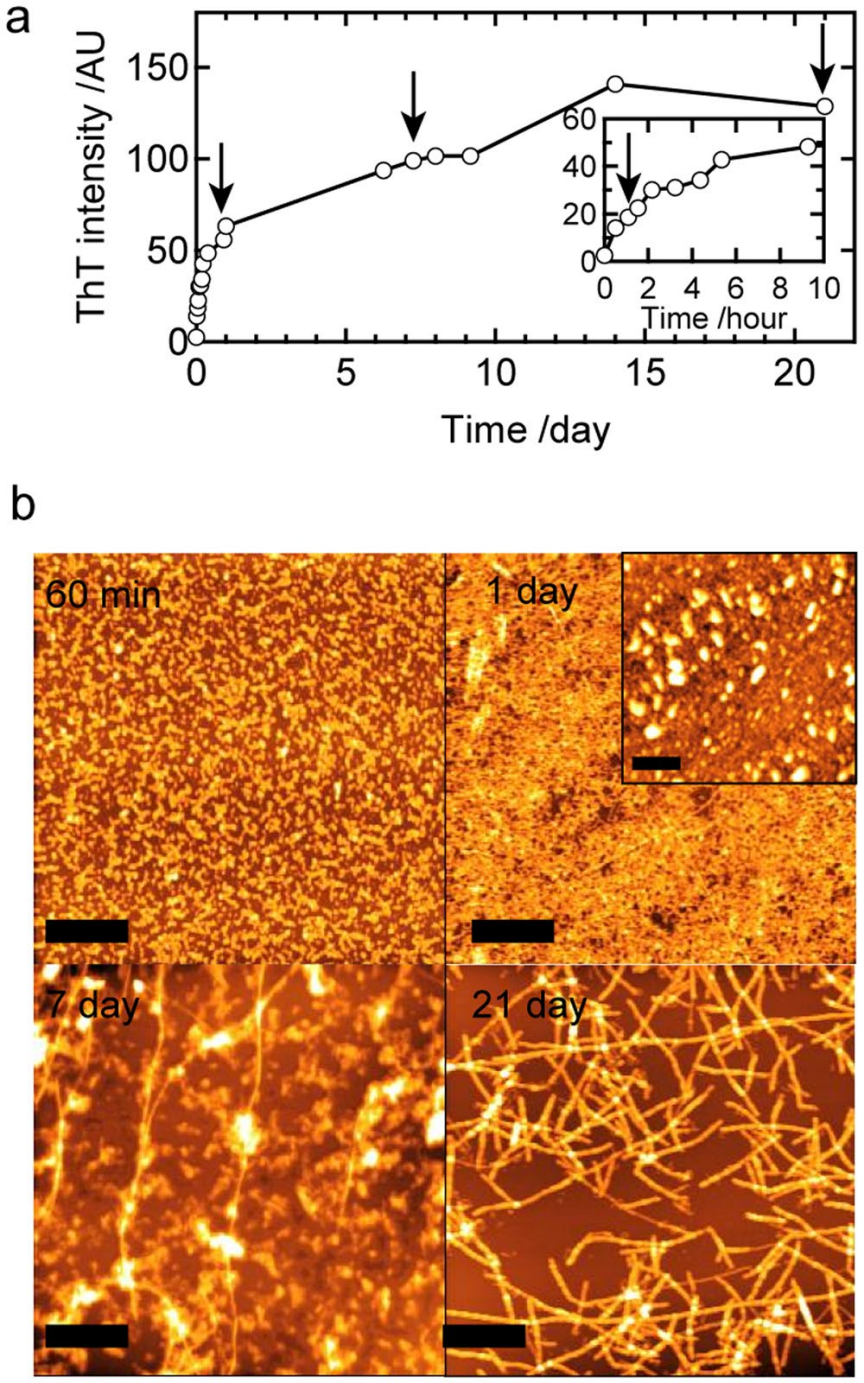
Time course of amyloid fibril formation by the B chain at pH 8.7 under non-agitated conditions. (**a**) Time course of ThT fluorescence intensity. A magnified view of the early time region is also shown as an inset. The arrows indicate time points where AFM samples were collected. (**b**) Corresponding time dependence of aggregation, as monitored by AFM images. The inset is an AFM image sampled without washing by water, which was observed at 510 min. The scale bars in AFM images represent 1 μm.

Interestingly, the later phase of the reaction, i.e., the formation of mature amyloid fibrils, was markedly retarded under quiescent conditions, i.e., without agitation (Fig. 3a). Within the 24-h incubation, no fibril-like structures were confirmed by AFM and only particles (Fig. 3b, 60 min) or aggregates of particles with a height of 3–5 nm (Fig. 3b, 1 day) were observed. Further incubations for days or weeks resulted in the appearance of fibril-like structures among aggregated particles (Fig. 3b, 7 days) and fibril-like structures eventually became the major products (Fig. 3b, 21 days). The results demonstrated that the lifetime of the prefibrillar intermediates significantly prolonged compared to that under agitated conditions. We confirmed that this increase pattern in the ThT intensity was reproducible (Fig. S1b).

**Figure 4:**
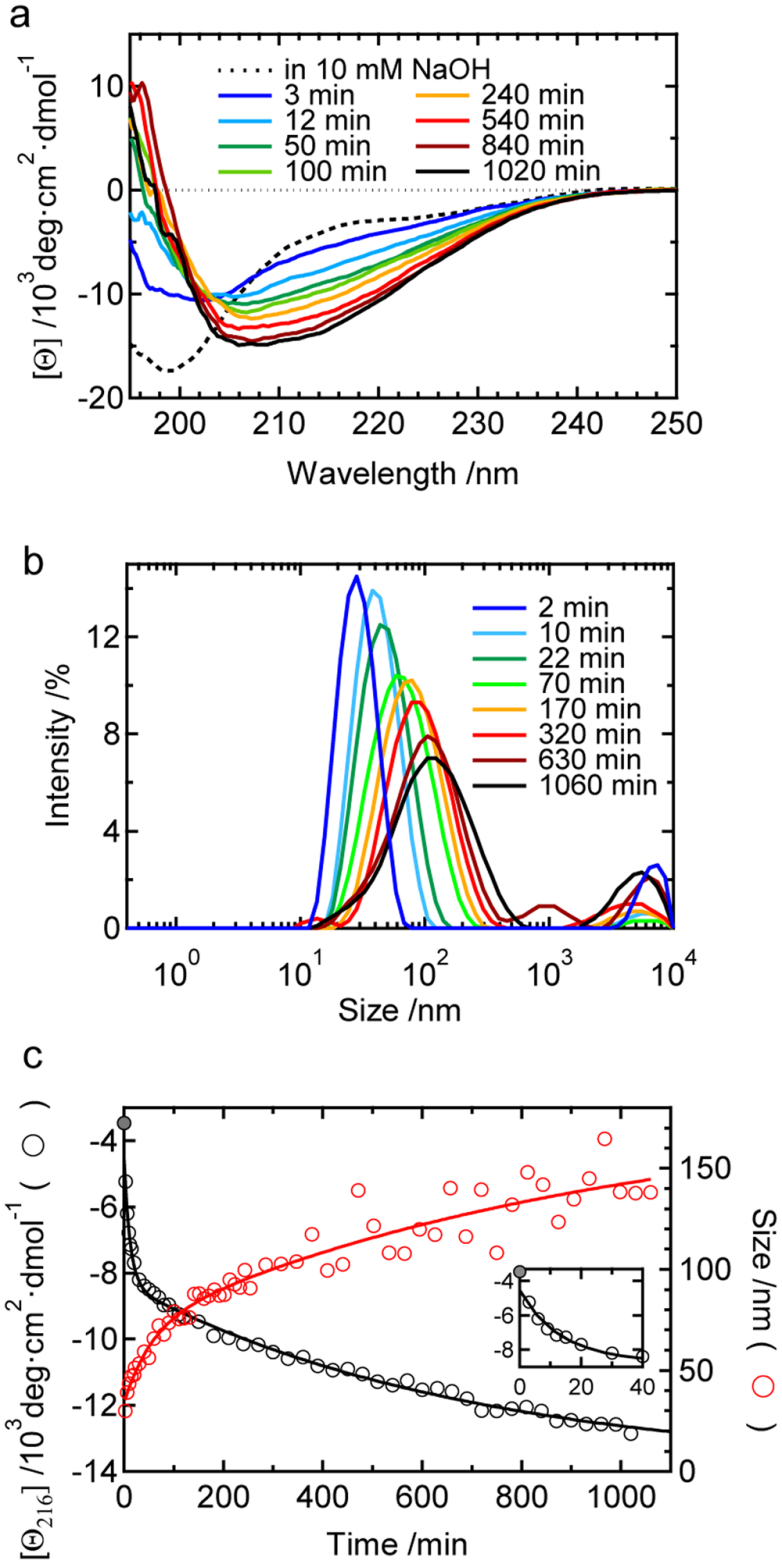
Formation and time evolution of the structure and size of prefibrillar aggregates as monitored at pH 8.7 under non-agitated conditions. Time-dependent changes in (**a**) CD spectra and (**b**) DLS size distributions. In panel a, the spectrum obtained in 10 mM NaOH is also shown by a dashed line as a reference. (**c**) Time course of molar ellipticity at 216 nm ([*θ*_216_]) and the position of the main peak in the size distribution of DLS. The black and red continuous lines show the fit curves obtained using equations 2 or 5, respectively. The inset shows a magnified view of the early time of the CD time-course plot.

We then investigated the early stage of quiescent conditions using CD spectroscopy and dynamic light scattering (DLS) to characterize the formation process of prefibrillar intermediates (Fig. 4). The sample was kept almost transparent during the time course, which enabled *in situ* monitoring. One example of the time courses of CD spectra is shown in Fig. 4a. The overlaid CD spectra showed two different isoellipticity points initially at approximately 204 nm and then at approximately 201 nm, suggesting that the reaction proceeds in a two-step manner. In accordance with this result, the time dependence of mean molar ellipticity at 216 nm ([*θ*_216_]) appeared to possess two phases: ellipticity markedly decreased within several dozens of minutes, and the slope of the decrease then became less steep (Fig. 4c, black plot). The time dependence was fit using a biexponential function described by eq. 2 (Materials and Methods). Based on three independent experiments, we obtained *τ*_1_ = 16 ± 2 min and *τ*_2_ = 530 ± 80 min with [*θ*]_0_, [*θ*]_1_, and [*θ*]_2_ of −4,300 ± 200, −7,000 ± 700, and −11,600 ± 1,200 deg·cm^2^·dmol^−1^, respectively (average ± S.D., *n* = 3). An additional measurement of attenuated total reflection-Fourier transform infrared (ATR-FTIR) spectra indicated a characteristic β-sheet band, suggesting that β-sheet structures predominantly developed to produce prefibrillar aggregates (Supplementary Fig. S2).

One example of the time courses of DLS results is shown in Fig. 4b. Intensity-based size distributions obtained by the CONTIN method with the data set of the autocorrelation function were used for the analysis. Within the time range of the measurement, one main peak was only obtained at each time point, except for a fraction of larger aggregates. The mean value of the hydrodynamic diameter (*D*_h_) of the main component (Fig. 4c, red plot) shifted in a time-dependent manner, which was also fit using a biexponential function described by eq. 5 (Materials and Methods). Based on three independent experiments, the values of *τ*_1_ and *τ*_2_ were 23 ± 15 min and 520 ± 210 min, respectively, with *D*_h0_, *D*_h1_, and *D*_h2_ of 24 ± 6, 70 ± 0, and 130 ± 30 nm, respectively (average ± S.D., *n* = 3). As for the values of *τ*_1_ and *τ*_2_, it should be noted that they are overestimated given that scattering intensity is proportional to the sixth power of the radius, whereas weight fraction as represented by molar ellipticity should be proportional to the cube of radius. However, the values of fast and slow time constants were in a good agreement with those obtained in the analysis of CD spectra, which indicates the presence of two distinct intermediate states in terms of structural order and aggregate size. The observation of only one main peak may have been due to small differences in the mean sizes of the two major prefibrillar aggregates in addition to their polydispersity, which may have made it difficult to distinguish them using the CONTIN analysis. Additionally, the particle sizes of the two intermediate species observed by DLS appeared to be markedly larger than the heights of particles confirmed in AFM images; when we prepared AFM samples without washing with water, large particles of 50–100 nm in diameter were confirmed in the image (Fig. 3b, inset), suggesting that prefibrillar intermediates are fragile and unstable against washing. Based on the CD and DLS results, two prefibrillar aggregate species were identified in the pathway of amyloid fibril formation, and we defined the first and second species as 1st and 2nd prefibrillar intermediates, respectively.

**Figure 5:**
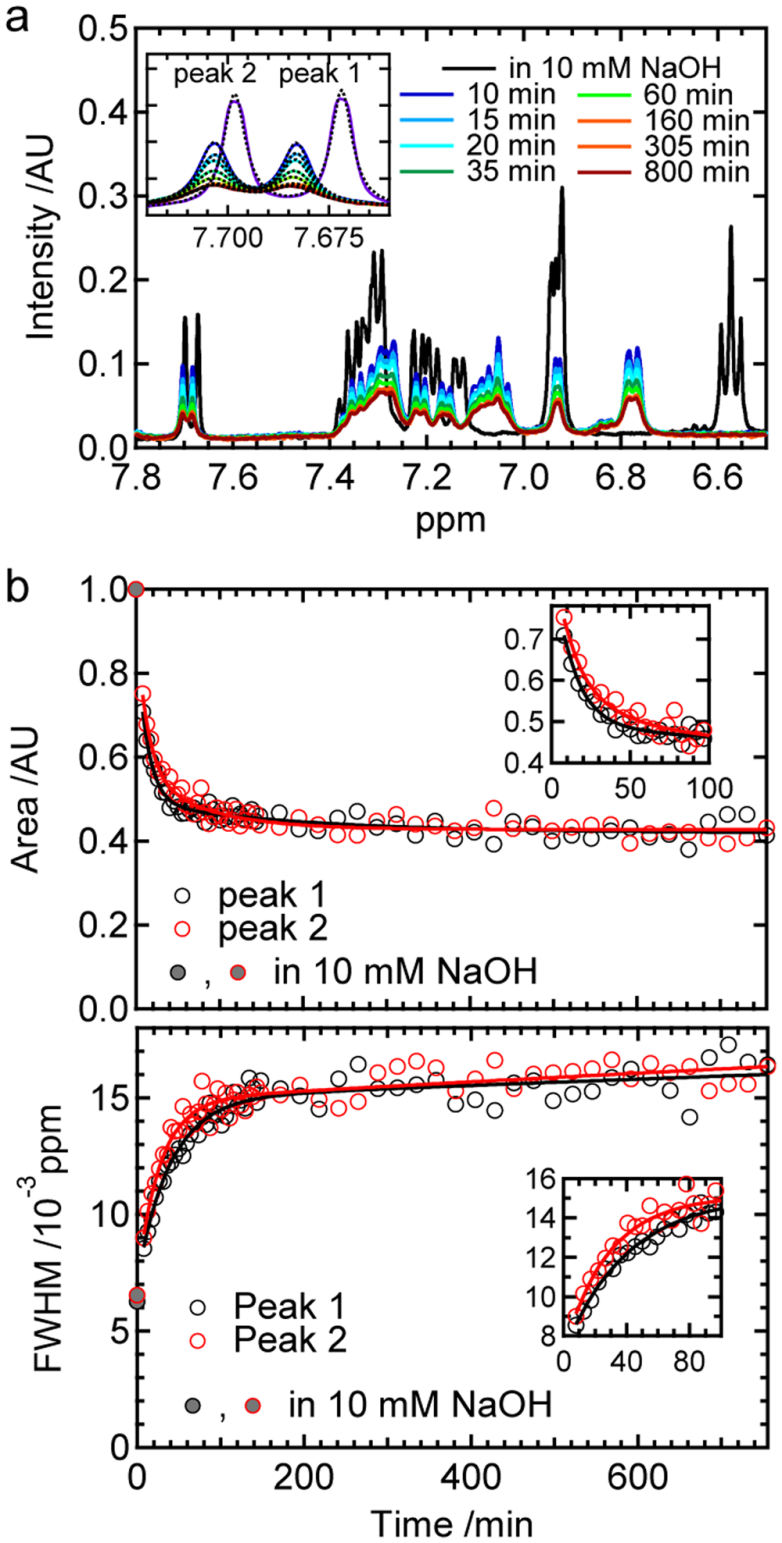
^1^H-NMR analysis of the formation and time evolution of prefibrillar aggregates. (**a**) Time-dependent ^1^H-NMR spectra in a low magnetic-field region obtained at pH 8.7 under non-agitated conditions. The spectrum taken in 10 mM NaOH is also shown by a purple line. The inset shows a magnified view of two histidine ε protons, which are termed as peak 1 and 2, respectively. Dashed lines show the fit curves obtained using equation 6. (**b**) Time-dependent changes in the areas (top) and FWHMs (bottom) of peaks 1 and 2 obtained by spectral fitting. In both panels, values of areas and FWHMs observed in 10 mM NaOH are also represented as filled circles as references. The results of curve fitting assuming a biexponential function are shown by continuous lines. The insets show the magnified figures in the early time period. Detailed information on fitting equations and the resulting values of parameters are summarized in Supplementary Results.

The stepwise formation process of prefibrillar intermediates was verified by measuring ^1^H-NMR spectra, which are sensitive to particle sizes and structures. After the reaction started, resonance peaks started to broaden with decreases in peak areas (Fig. 5a), indicating that the apparent molecular weight of the B chain increased as a function of time in conformity with DLS results. Focusing on two well-isolated peaks originating from *ε* protons in the histidine residues of the B chain peptide (Fig. 5a, inset), their area and peak width were plotted against time, and fit using a biexponential function (Fig. 5b; see also Supplementary Results for details). The time constants obtained from the peak areas of peak 1 and 2 were *τ*_1_ = 13 ± 2 min and *τ*_2_ = 200 ± 110 min and *τ*_1_ = 14 ± 5 min and *τ*_2_ = 80 ± 30 min, respectively. These values are fairly in good agreement with the time constants obtained by the CD and DLS analyses, confirming the two-step development of prefibrillar intermediate species. The analysis of the time course of the full width of half maximum (FWHM) of these two peaks also showed a similar result to that of the peak areas (see Supplementary Results). In a rough estimation, a proton signal typically disappears when the molecular diameter becomes 30–40 nm^21^. The DLS-estimated diameters of the 1st and 2nd intermediates, i.e. ~70 and ~130 nm, are beyond the detection limit of liquid-state NMR, which reasonably explains decreases in the peak areas. It is important to note that peak areas did not reach zero after the reaction reached equilibrium (Fig. 5b), which indicates that significant amounts of NMR-detectable monomers co-exist, even after the formation of prefibrillar intermediates.

### Ultrasonication-enhanced formation of amyloid nuclei from prefibrillar intermediates

The contrastive monitoring of fibrillation reactions under agitated and non-agitated conditions suggested that agitation enhances the structural conversion of prefibrillar intermediates to produce amyloid nuclei. To assess this possibility, we analyzed an effect of transient agitation on the structures of prefibrillar intermediates. In this analysis, we focused on ultrasonication as an alternative to the shaking method in order to take advantage of the high efficiency to enhance the spontaneous formation of amyloid fibrils^22–25^ in addition to easy-to-control power output and durations. The sample was exposed to the ultrasonic wave with an output of 2 watts for 1 sec, immediately after which the effect of the ultrasonic treatment was tracked using CD spectra under quiescent conditions. We confirmed that the temperature increase by the ultrasonic treatment was less than 1 °C, and no change in the particle size was observed based on DLS measurements (Supplementary Fig. S3), verifying that the output level was not sufficiently large to destroy prefibrillar intermediate structures.

**Figure 6:**
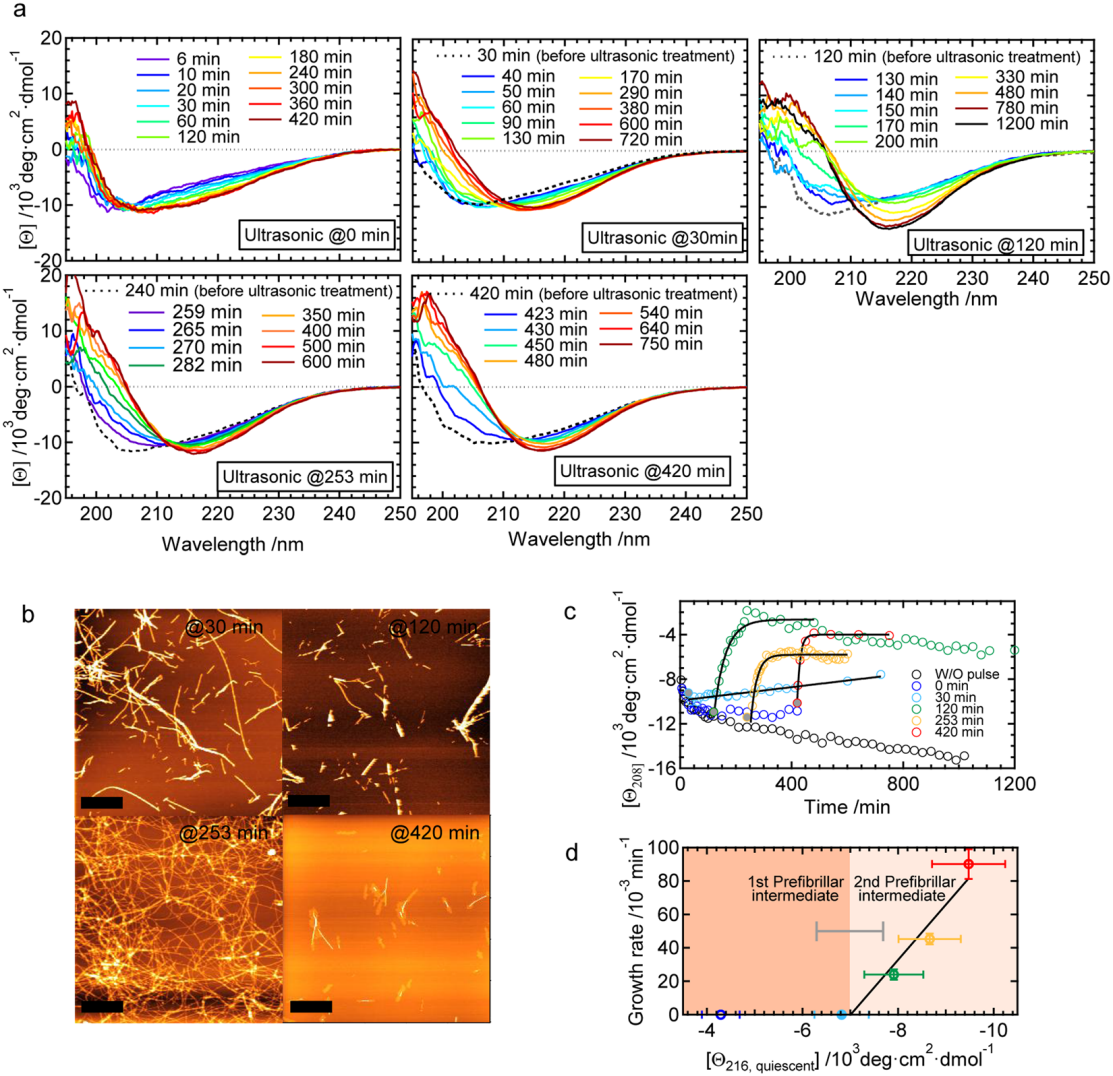
Effects of the ultrasonic pulse treatment on prefibrillar intermediates. (**a**) Time-dependent changes in CD spectra after the ultrasonic treatment. The results of five different timings of the ultrasonic treatment are shown. Except for the panels of ultrasonication at 0 min, CD spectra measured before the ultrasonic treatment are also shown by dashed lines as references. (**b**) AFM images of final reaction products obtained by analyses of the ultrasonic treatment at 30, 120, 253, and 420 min. The scale bars in AFM images indicate 1 μm. (**c**) Summary of the time dependency of CD spectral changes after the ultrasonic treatment, which was represented by plotting molar ellipticity at 208 nm ([*θ*_208_]) against time. Values without the ultrasonic treatment (W/O pulse) were taken from the data shown in Fig. 4a. The black continuous lines show fit curves assuming a single exponential function. (**d**) Relationship between the fibril growth rate obtained by curve fitting in panel c and the molar ellipticity at 216 nm just before the ultrasonic treatment ([*θ*_216, quiescent_]). The growth rate observed by the 0-min treatment was defined as zero because of the lack of fibril growth within our observation time. Two phases for the 1st prefibrillar intermediate and 2nd prefibrillar intermediate are colored separately based on the asymptotic values of molar ellipticity (i.e. [*θ*]_1_ and [*θ*]_2_), which were obtained by the curve fitting of the CD time-course measurements in the quiescent conditions. The error bars were obtained by calculation based on the standard deviations for the three independent measurements.

An ultrasonic wave was applied after the time period spared for the formation of prefibrillar intermediates (i.e., 30 min, 120 min, 253 min, and 420 min). As a result, CD spectra shifted towards a typical spectrum for β-sheet structures with a minimum at approximately 216 nm (‘ultrasonic @ 30/120/253/420 min’ in Fig. 6a). These results suggest that the ultrasonic wave induced the amyloid fibril formation, which was confirmed in AFM images (Fig. 6b). In contrast to these results, when the ultrasonic pulse was applied immediately after the preparation of the peptide solution where monomeric species were expected to be dominant, CD spectra showed a similar time dependence to that of the formation of prefibrillar intermediates (‘ultrasonic @ 0 min’ in Fig. 6a; see also Fig. 4a). These results demonstrate that the ultrasonic pulse-induced formation of mature amyloid fibrils is only achieved in the presence of prefibrillar intermediates.

To evaluate the kinetics of β-sheet development, mean residue ellipticity at 208 nm, which markedly increased with the β-sheet formation, was plotted, as shown in Fig. 6c. Here, we faced difficulty in monitoring structural changes at 216 nm because of small changes in signal intensities, and thus we chose 208 nm instead of 216 nm. Values started to increase immediately after the ultrasonic treatment, except in the case of the 0-min treatment. Time-dependent changes in CD values were simulated by a single exponential curve assuming that pseudo-first-order kinetics, which is typically observed in seed-dependent elongation reactions, was valid. The extrapolation of theoretical curves obtained by the curve fitting almost crossed the values before the ultrasonic treatment in all cases (filled circles in Fig. 6c), demonstrating the absence of any lag phase. This result strongly suggests that the amyloid-like structure that functions as nuclei already came into existence promptly after the exposure of prefibrillar intermediates to the ultrasonic wave. Although we expected to detect some structural changes after the ultrasonic treatment, any significant indication of the nuclei formation was not found based on the CD and DLS measurements (Fig. 6 and Supplementary Fig. S3), suggesting that only a fractional change occurred during nucleation. When the growth rate of amyloid fibrils was plotted against the value of mean residue ellipticity at 216 nm just before applying the ultrasonic pulse ([*θ*_216,quiescent_]), a good linear relationship was revealed (Fig. 6d). The intersection point of the abscissa axis, ~−7,000 deg·cm^2^dmol^−1^, was in good agreement with the point at which the first exponential decay was completed and the second decay started (i.e., −7,000 deg·cm^2^dmol^−1^; see Fig. 4c). These results strongly indicate that the 2nd prefibrillar intermediate, not the 1st is susceptible to the ultrasonic treatment to produce amyloid nuclei.

## Discussion

### Amyloid nucleation of the B chain associated with a specific prefibrillar aggregate species

We identified and characterized prefibrillar aggregates of the insulin B chain peptide that preceded the formation of mature amyloid fibrils. Due to their metastable characteristics under quiescent conditions, we successfully tracked time evolution in detail and revealed two major species, i.e. the 1st and 2nd prefibrillar intermediates. An important feature of these prefibrillar aggregates is that they easily transformed into the most stable fibrils once agitation was supplied, probably due to a moderate height of the energy barrier between the prefibrillar and fibrillar states. As a result of this property, the fibril formation was triggered even with the application of one short ultrasonic pulse to the intermediates. Furthermore, by investigating the relationship between the efficiency of amyloid fibril formation and the timing of ultrasonic triggering, we demonstrated that a specific form of prefibrillar aggregates has the potential to become amyloid nuclei.

pH 8.7 is a slightly alkaline point from the isoelectric point of B chain, pH 7.8, where the side-chain thiol groups of two cysteine residues, Cys7 and Cys19, are considered to be additionally deprotonated as compared to the state at the isoelectric point. Given that severe peptide aggregation rapidly occurred close to the isoelectric point, the introduction of a slight amount of charge repulsion between side chains would have prevented amorphous aggregation. It is suggested that a balance between the electrostatic and hydrophobic interactions determines the fate of protein aggregation, i.e. the formation of amyloid fibrils or amorphous aggregation^26^. Similarly, one possible scenario in this study is that a subtle balance between charge repulsions and van der Waals interactions would have led to the formation of moderately organized prefibrillar intermediates.

**Figure 7:**
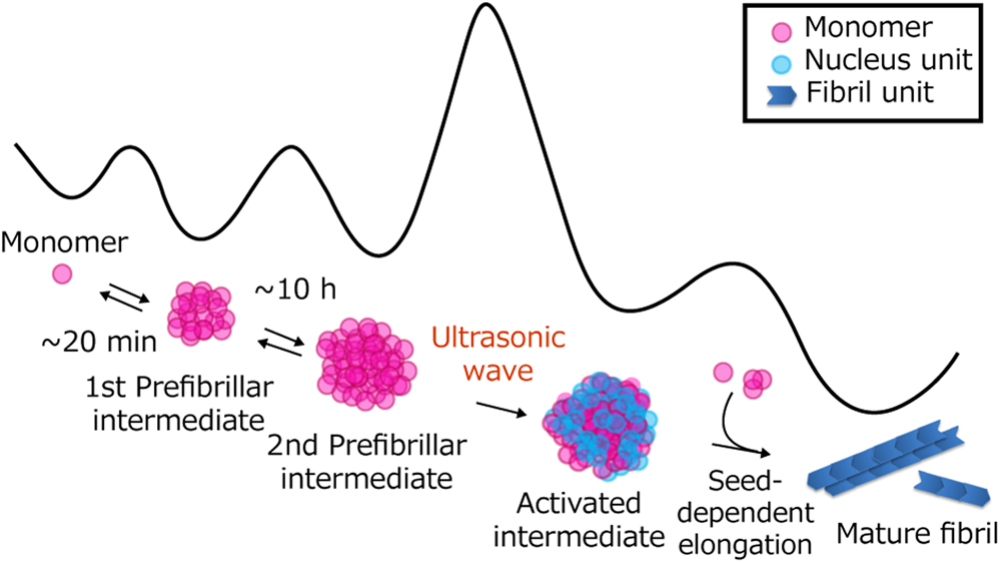
Schematic drawing of the B chain fibril formation involved in multi-step nucleation via a specific prefibrillar intermediate. The structural illustrations for molecular species that are detected or predicted in this study are drawn with an overall shape of the energy surface deduced for the fibril formation reaction at pH 8.7. Note that the shape and energy level of each molecular species and the height of the energy barrier in this illustration are tentative and do not include quantitative information.

Figure 7 shows a schematic drawing of the sequential development of the size and β-sheet structure of the insulin B chain during the fibrillation reaction at pH 8.7. The 1st prefibrillar intermediate is initially formed, and this is followed by the accumulation of the 2nd prefibrillar intermediate, which has been found under quiescent conditions. The highest energy barrier in the whole process is located between the 2nd prefibrillar intermediate and the state of amyloid nuclei because amyloid nucleation was triggered by an ultrasonic pulse only after the formation of the 2nd prefibrillar intermediate (Fig. 6d). Interconversions among molecular species from the monomer to 2nd prefibrillar intermediate appear to be reversible (equilibrium arrows in Fig. 7) based on the time-resolved ^1^H-NMR measurement (Fig. 5b). Once B chain peptides exceed the energy barrier with the aid of an external force and amyloid nuclei form, amyloid fibrils grow via a mechanism known as seed-dependent elongation (the rightmost arrow in Fig. 7)^27^. Since the particle sizes of prefibrillar intermediates are clearly larger than the diameters of amyloid fibrils, the elongation step of amyloid fibrils appears to proceed in parallel with the dissociation of prefibrillar intermediates to smaller monomers or oligomers. The mechanistic scheme of ‘nucleation from prefibrillar aggregates’ deduced by the present study may also explain previous findings showing that a long and thin amyloid fibril was germinated from the granular particles of aggregates^28,29^.

Ultrasonication was originally used to fragment long fibrils in the preparation of seed fibrils and has since become a powerful driving force to induce the formation of amyloid fibrils^22,23,25^. The ultrasonic irradiation of liquids induces the formation of transient microbubbles that then collapse, during which the local temperature is proposed to transiently increase^25,30^. Under these basic mechanisms, proteins or peptides are more likely to be condensed and partially denatured on the surface of microbubbles, and amyloid nucleation may then be accelerated by microbubbles colliding together and the large increase in temperature associated with the collapse of microbubbles. In view of the present results showing that fibril formation was also accelerated by shaking (Fig. 2), the external mechanical force generated at the air-water interface was sufficient to change the intermolecular interaction of peptide structures in the 2nd prefibrillar intermediate to that of nuclei. The 2nd prefibrillar intermediate is also predicted to be formed in the reaction with continuous shaking; however, its amount appeared to be smaller than that under quiescent conditions because the mean size obtained by DLS was ~70 nm at the beginning of the later phase (Supplementary Fig. S4), which was almost the same size as that of the 1st prefibrillar intermediate observed under quiescent conditions (Fig. 4b). In this case, continuous agitation may have further accelerated the subsequent elongation step by enhancing the fragmentation of amyloid fibrils formed and/or secondary nucleation on the surface of fibrils, even though the initial amount of amyloid nuclei generated was small.

### Role of prefibrillar intermediates in amyloid nucleation

Based on the linear relationship between the amount of the 2nd prefibrillar intermediate and that of the nuclei produced upon the ultrasonic treatment (Fig. 6d), the nucleation of insulin B chains occurs via a multistep scheme, not a simple one-step one. Although no trace of conformational changes during nucleation was detected in the present study, the instant formation of amyloid nuclei after the ultrasonic pulse suggests that nucleation proceeded within the 2nd prefibrillar intermediate following a nucleated-conformational-conversion (NCC) mechanism, in which early aggregates are directly converted to amyloid nuclei. Since its first proposal in 2000^31^, the NCC mechanism has been supported by studies demonstrating the presence of on-pathway prefibrillar intermediates^5,8,9,32^. Furthermore, several techniques including ion mobility-mass spectrometry^10^, ESR^12^, time-lapse solid-state NMR^11^, and the development of methodology using hybrid peptides^33^ or a latent fluorophore^34^ recently revealed the successful conformational conversion of aggregates to β-sheet-rich fibrils. Previous finding also showed that aggregates convert to mature amyloid fibrils in parallel with their mutual association^35^, which may also be categorized as the NCC mechanism in a broad context.

The advantage of forming intermediate aggregates prior to nucleation is an increase in local peptide concentrations and, hence, chances to form intermolecular interactions required for fibril formation become larger than those without these aggregates. Furthermore, permittivity inside aggregates is generally markedly lower than that of water. Such a hydrophobic environment may serve as a reaction field that facilitates the rearrangement of hydrogen bonds to form the β-sheet structures of nuclei. The likelihood of nucleation via prefibrillar aggregates has been theoretically proposed by a coarse-grained model simulation^36^. However, the ability of the 2nd prefibrillar intermediate to act as a nucleation-prone species, while the 1st prefibrillar intermediate does not act as well cannot be answered by the above explanation alone. In order to clarify the reason for this difference in susceptibility to nucleation, we attempted to focus on differences in the secondary structures of the 1st and 2nd prefibrillar intermediates; a singular value decomposition analysis was performed on the data set of the time course of CD spectra obtained under quiescent conditions (Fig. 4a). However, no trace of a difference in the secondary structure was revealed based on this analysis, and the β-sheet structure was consequently suggested to be fundamentally similar, although its content varies in these intermediates (Supplementary Fig. S5). Although we may only speculate on the underlying molecular mechanisms, the inclusion of more than a threshold amount of the β-sheet structure in aggregates may result in some characteristic structures, for example, the types of substructures that accelerate the formation of nuclei.

### Conclusions and implications for amyloid nucleation in biological systems

The molecular picture obtained in the present study has provided an important insight into amyloid formation that will promote our understanding of the significance of prefibrillar aggregates, particularly in nucleation. Since amyloidogenic proteins or peptides are mostly rich in hydrophobic amino acid residues, they appear to have the ability to form aggregates. Once these aggregates are formed, they are expected to produce amyloid nuclei more easily than an isolated ensemble of monomeric molecules because of greater chances to interact with each other. Recent studies on the crystallography of many inorganic and organic compounds described examples of the presence of aggregates called prenucleation clusters, which has indicated the need to consider more complex processes in addition to the classical nucleation theory^37^. When the present results are compared with these crystals, the 2nd intermediate of the B chain has a similar appearance to the prenucleation clusters, corroborating the deep involvement of prefibrillar aggregates in amyloid nucleation. It should be noted that not all nuclei responsible for amyloid fibril formation necessarily result from prefibrillar aggregates. In actual cases, the classical nucleation mechanism and NCC mechanism may co-exist. Moreover, down-hill fibrillation, in which no nuclei are required for fibril formation, has been reported for transthyretin^38^. The probable mechanisms vary depending on the types of proteins and peptides as well as the experimental conditions such as temperature, pH, and salt concentration.

Prefibrillar aggregates may also play an important role in fibril formation in *in vivo* systems. Individual protein concentrations in a living system are generally markedly lower than those conventionally used *in vitro*, and the reaction field is far from the ideal homogeneous system because proteins are surrounded by various biomacromolecules. Under these conditions, simple nucleation described by the standard classical nucleation theory rarely occurs. As an alternative for nucleation, other cellular components such as lipid membranes may induce the formation of aggregates similar to the 2nd prefibrillar intermediate of the B chain peptide^39,40^, which may then be converted to amyloid fibrils upon physical perturbations created by biomechanical forces.

A large number of experimental studies have examined various kinds of protein oligomers and aggregates, and whether they function as an on-pathway species undertaking the role as nucleation precursors, an off-pathway species, or dead-end species has been a controversial issue. Therefore, the present results clarified two contrastive types of prefibrillar aggregates, one of which contributes to nucleation, while the other does not, thereby providing an important clue for identifying key factors that dictate their ability to convert to nuclei. More specialized structural discriminations of the 1st and 2nd prefibrillar intermediates as well as the accumulation of studies on other amyloid fibril-prone proteins and peptides that focus on the characterization of prefibrillar intermediates will reveal the structural entities that serve as the source of amyloid nucleation. This will contribute to the development of preventive therapy for amyloid diseases by utilizing specific types of early aggregate responsible for nucleation as a target for the effective inhibition of amyloid nucleation, and, thereby, the prevention of pathogenesis *in vivo*.

## Materials and Methods

### Purification of B chain

Human insulin (Wako Pure Chemical Industries, Ltd., Japan) was dissolved in buffer solution composed of 50 mM Tris-HCl and 100 mM NaCl, the pH value of which was adjusted to 8.7. DTT solution was added to start the reduction of insulin, with adjustments in pH using HCl to 8.6. The final concentration of insulin was 5 mg/ml. Immediately after the addition of DTT, the solution became turbid because of the precipitation of the B chain. The solution was kept under 25 °C for 15 h. The precipitate was then separated by ultracentrifugation at 5,000 × *g* under 4 °C for 10 min. The precipitate was rinsed using cold Milli-Q® water (Merck Millipore Corp., Germany) and centrifuged in the same manner. The same washing protocol was repeated 4 times. The washed precipitate was dissolved in 10 mM NaOH to reach a B chain concentration of 2.5–3.5 mg/ml, frozen using liquid nitrogen, and stored at −80 °C. B chain concentrations were measured by using the absorption coefficient of 0.90 (mg/ml)^−1^cm^−1^ at 280 nm in NaOH solution. The purity of the B chain was more than 95%, as assessed by the ^1^H signals of ε protons in tyrosine residues obtained using the NMR spectrometer, AVANCEIII HD (Bruker, Germany).

### Formation of B chain amyloid fibrils

The purified B chain stocked in 10 mM NaOH was diluted by an approximately half volume of various buffer solutions to reach a final concentration of 1.4 mg/ml. pH values were adjusted using two types of 50 mM buffers: acetate-sodium acetate for pH 5.0, and Tris-HCl for pH 8.7. HCl was used to directly adjust pH to 1.6. In the time-course experiments performed at pH 8.7, a B chain stock solution in NaOH at a concentration of 2.8 mg/ml was diluted by the same volume of buffer solution in order to reach Tris-HCl and NaCl final concentrations of 50 mM and 5 mM, respectively. As for chemical stability of the B chain peptide, ^1^H-NMR spectra measured at a low B chain concentration, where no aggregation occurred, showed no remarkable changes as far as 24 hours at pH 8.7 and 25 °C.

All experiments were performed at 25 °C, and, in experiments conducted under quiescent conditions, B chain solutions were placed in plastic tubes and the lids were carefully closed to prevent air-water interfaces. Experiments under agitated conditions were also performed under continuous shaking using the mixer, ThermoMixer C (Eppendorf, Hamburg, Germany) at 1,200 rpm. In ultrasonication-induced fibrillation experiments, an ultrasonic wave with a frequency of 22.5 kHz was directly applied to the solution through the tip of the ultrasonic liquid processor, MICROSON^TM^ XL 2000 (Qsonica, LLC., CT). The duration of the ultrasonic wave was 1 sec at an output power of 2 watts.

### ThT assay

The formation of aggregates or amyloid fibrils was monitored using the fluorescent dye, ThT. In the assay, 5 μl of a sample solution was mixed with 1.5 ml of a ThT assay solution, which was composed of 5 μM ThT and 50 mM glycine-HCl (pH 8.5). One minute after the incubation, a fluorescent spectrum was recorded between 430 and 530 nm with excitation at 445 nm with a high scan speed using the spectrofluorometer, RF-5300pc (Shimazu Co., Ltd, Japan). The bandwidth for excitation and emission was set to 5 nm.

### AFM

Five or ten microliters of a sample were loaded to a mica plate, left for one minute, and then rinsed using 1 ml of Milli-Q water. We used this normal sample preparation method unless otherwise indicated. We also skipped the rinsing process in order to prevent the disruption of prefibrillar intermediates (see Results). AFM images were obtained using the dynamic force mode with Probestation NanoNavi II/IIe (SII Nanotechnology, Japan). The sweep rate was set to 0.5 or 1.0 Hz with the recording of 256 × 256 points per image.

### CD spectroscopy

CD spectra were obtained using the CD spectrometer, J-720 or J-1100 (JASCO, Japan). Liquid samples were placed in a quartz cell with a path length of 0.2 mm. Each scan was performed at 100 nm/min or 200 nm/min from 250 to 195 nm, and two or four individual scans were integrated and averaged to obtain one spectrum. The mean residue molar ellipticity, [*θ*], which is molar ellipticity normalized by the number of amino acid residues, was calculated as follows:

1 $$[\theta ]=\frac{100\theta }{cl{N}_{{\rm{aa}}}}/{\rm{\deg }}\cdot {{\rm{cm}}}^{2}\cdot {{\rm{dmol}}}^{-1}$$

where *θ*, *c*, *l*, and *N*_aa_ represent experimentally-obtained ellipticity (in deg), the molar concentration of a sample (in M), the path length of a cell (in cm), and the number of amino acid residues, respectively. A time-course in a quiescent condition was fit using a biexponental equation below;

2 $$[\theta ](t)={[\theta ]}_{2}-({[\theta ]}_{1}-{[\theta ]}_{0})\exp (-\frac{t}{{\tau }_{1}})-({[\theta ]}_{2}-{[\theta ]}_{1})\exp (-\frac{t}{{\tau }_{2}})$$

where *τ*_i_ and [*θ*]_i_ represent the apparent time constants of the ith phase and the asymptotic value of molar ellipticity after the completion of the ith phase, respectively.

### DLS

In order to estimate particle sizes, DLS experiments were performed using Zetasizer Nano-S (Malvern Instruments, Worcestershire, UK). A He-Ne laser at a wavelength of 633 nm was introduced to a sample cuvette and back scattered light was detected using an avalanche photodiode at a scatter angle of 173°. Scattered photons were corrected for 10 seconds and an autocorrelation function of fluctuations in the intensity of the scattered laser was obtained. The autocorrelation function of the correlation time *τ* is described as follows:

3 $${g}^{(2)}({\bf{q}},\tau )=\frac{\langle I({\bf{q}},0)I({\bf{q}},\tau )\rangle }{{\langle I({\bf{q}},0)\rangle }^{2}}$$

where *I*(**q**, *τ*) represents the intensity of the scattered laser as a function of a scattering vector, **q**. The absolute value of **q**, i.e. $|{\bf{q}}|(\equiv q)$ is $q=\frac{4\pi n}{{\lambda }_{0}}\,\sin (\frac{\theta }{2})$ where *n*, *λ*_0_, and *θ* indicate the refractive index of the sample, the wavelength of the He-Ne laser, and the scatter angle, respectively. This scan was repeated as judged by the controlling software, and scans were then averaged and used to analyze particle sizes. Size distribution was obtained using the CONTIN method, in which the autocorrelation function was analyzed using a multiexponential function to obtain the diffusion coefficient^41^. Assuming that a particle is spherical, the hydrodynamic diameter *D*_h_ is described by the Stokes-Einstein relationship:

4 $${D}_{{\rm{h}}}=\frac{{k}_{{\rm{B}}}T}{3\pi \eta D}$$

where *T*, *η*, and *D* represent the temperature, viscosity of the system, and diffusion coefficient, respectively. The parameters used for the calculation were as follows: *n* = 1.331, *λ*_0_ = 633 nm, *θ* = 173 °, *k*_B_ = 1.386 × 10^−23^ J/K, *η* = 8.93 × 10^−4^ Pa·s, and *T* = 298.15 K. For the analysis of particle size, intensity-based size distributions were used. Data collection and processing were performed using the software, Dispersion Technology Software 5.00 (Malvern Instruments Ltd., UK). A time-course of the hydrodynamic diameter in a quiescent condition was fit using an equation below;

5 $${D}_{{\rm{h}}}({t})={{D}}_{{\rm{h}}2}-({{D}}_{{\rm{h}}1}-{{D}}_{{\rm{h}}0})\exp (-\frac{{t}}{{\tau }_{1}})-({{D}}_{{\rm{h}}2}-{{D}}_{{\rm{h}}1})\exp (-\frac{{t}}{{\tau }_{2}})$$

where *τ*_i_ and *D*_hi_ represent the apparent time constants of the ith phase and the asymptotic value of hydrodynamic diameter after the completion of the ith phase, respectively.

### ^1^H-NMR spectroscopy

NMR spectral measurements were performed using AVANCE III HD equipped with a superconducting magnet that had a Larmor frequency of ^1^H of 400.13 MHz (Bruker, Germany). The spectrometer was controlled using the program, Topspin 1.5 (Bruker, Germany). The routine tool Topshim built in Topspin 1.5 was performed to achieve high homogeneity of the magnetic field. The p3919gp pulse program, which was pre-installed in Topspin, was employed to suppress the water signal using the WATERGATE pulse sequence^42^. A total of 800 μl of a sample was placed in a glass tube with an inner diameter of 4.95 mm, and all experiments were performed at 25 °C. In the fitting of a set of two proton peaks originating from the ε protons of histidine residues in the B chain peptide, a sum of two Lorentzians was used as follows:

6 $$I(\delta )=\sum _{{\rm{k}}=1}^{2}\frac{{{a}}_{{\rm{k}}}}{{(\delta -{\delta }_{{\rm{k}}})}^{2}+{\gamma }_{{\rm{k}}}}$$

where *I*(*δ*) is peak intensity at the frequency *δ*, and *a*_k_, *δ*_k_, and *γ*_k_ represent the amplitude, center frequency, and damping factor of the NMR peaks being focused on. The peak area and full width at FWHM were described as π*a*_k_/*γ*_k_ and 2*γ*_k_, respectively.

### Data Availability Statement

All data generated or analysed during this study are included in this published article (and its Supplementary Information files).

## Electronic supplementary material

Supplementary Information
