# Supplementary material for: A specific form of prefibrillar aggregates that functions as a precursor of amyloid nucleation
Source: Sci Rep. 2018 Jan 8;8:62. doi: 10.1038/s41598-017-18390-y (PMC5758805; doi:10.1038/s41598-017-18390-y)
Supplement: Supplementary file 1 — Supplementary Information [file 41598_2017_18390_MOESM1_ESM.pdf]

Supplementary Results and Figures

A specific form of prefibrillar aggregates that functions as a precursor of amyloid nucleation

Naoki Yamamoto, Shoko Tsuhara, Atsuo Tamura, and Eri Chatani

Supplementary Results

Curve fitting of the time dependence of area and FWHMs of two ^{1}H-NMR peaks of histidine ε protons in the B chain

The decay curves in Fig. 5b were fit using a double exponential function. The equation used to fit the peak area in Fig. 5b was as follows:

$$A(t) = (A_0 - A_1)\exp\left(-\frac{t}{\tau_1}\right) + (A_1 - A_2)\exp\left(-\frac{t}{\tau_2}\right) + A_2 \qquad \text{(S1)}$$

where τ_i and A_i represent the apparent time constants of the ith phase and the asymptotic value of peak area after the completion of the ith phase, respectively. In the case of peak 1, τ_1 and τ_2 were 13±2 min and 200±110 min, respectively, with A_0, A_1, and A_2 = 0.89, 0.49, and 0.42, respectively. In the case of peak 2, τ_1 and τ_2 became 14±5 min and 80±30 min, respectively, with A_0, A_1, and A_2 = 0.92, 0.57, and 0.43, respectively.

The equation used to fit FWHMs in Fig. 5b was as follows:

$$F(t) = F_2 - (F_1 - F_0)\exp\left(-\frac{t}{\tau_1}\right) - (F_2 - F_1)\exp\left(-\frac{t}{\tau_2}\right) \qquad \text{(S2)}$$

where τ_i and F_i represent the apparent time constants of the ith phase and the asymptotic value of FWHM after the completion of the ith phase, respectively. In the case of peak 1, τ_1 and τ_2 were 10±20 min and 55±12 min, respectively, with F_0, F_1, and F_2 = 6.5×10^{-3}, 8.6×10^{-3}, and 15.6×10^{-3} ppm, respectively. In the case of peak 2, τ_1 and τ_2 were 21±5 min and 190±110 min, respectively, with F_0, F_1, and F_2 = 6.5×10^{-3}, 13.9×10^{-3}, and 16.1×10^{-3} ppm, respectively. These time constants were qualitatively similar to those of the CD and DLS analyses, supporting the two-step development of prefibrillar intermediate species.

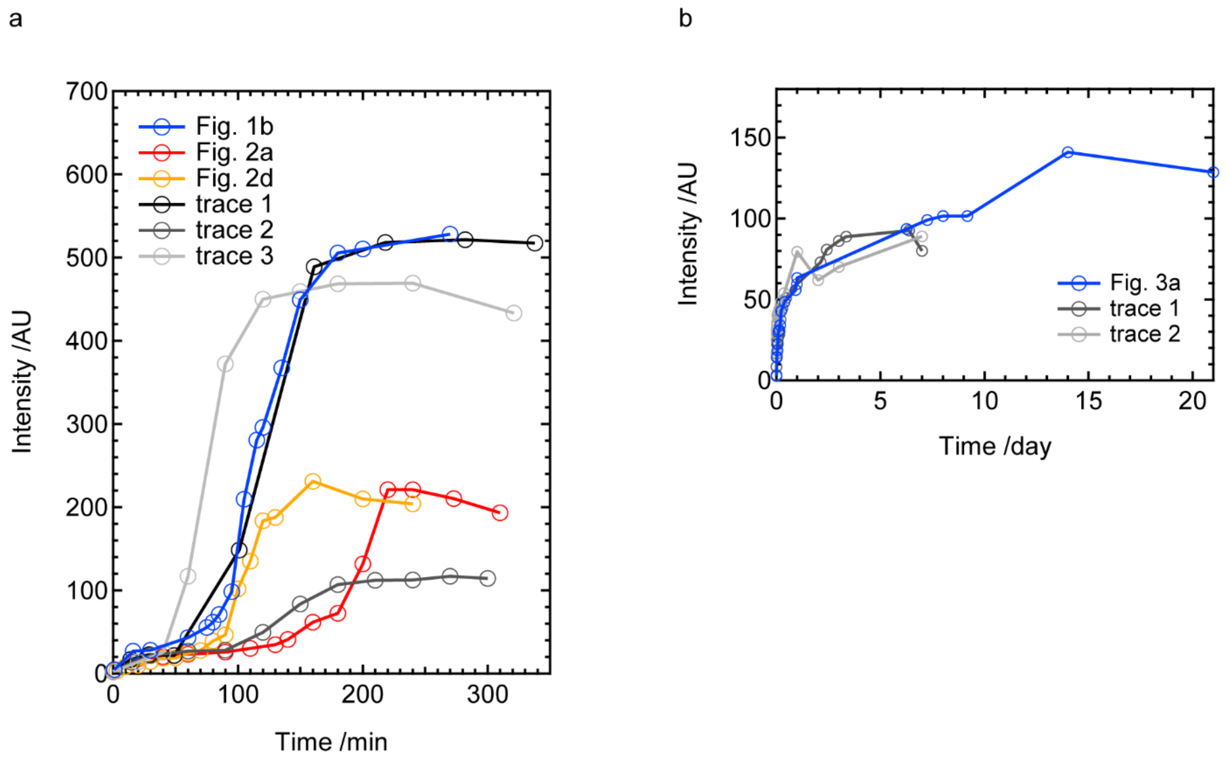

Figure S1. ThT time-courses under agitated and quiescent conditions. Several individual experiments were repeated at the B-chain concentration of 1.4 mg/ml under the condition of pH 8.7 (**a**) under agitated and (**b**) quiescent conditions. Traces used in the main text were colored and indicated in the legends. As shown in panel a, the 1st increase in the ThT intensity was always observed. This represents that the formation of the prefibrillar aggregates commonly occurs. On the other hand, the time when the 2nd increase started varied from 40 to 130 min, and moreover, the ThT fluorescent value in the plateau point also differed among the experiments. This result suggests that it is difficult to control the timing of the fibril formation and final ThT intensity strictly, as have often been observed in amyloid fibril formation reported in literature[1-4]. Nevertheless, all the traces possessed clear two-phase increase, verifying possible formation of prefibrillar intermediates.

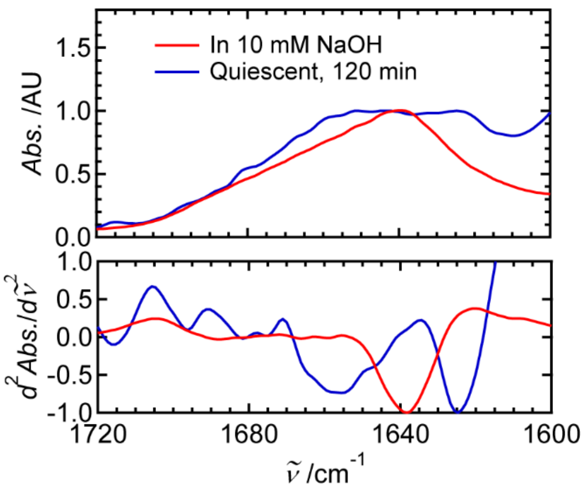

Figure S2. FTIR absorption spectra at the amide I region of prefibrillar aggregates. Since it was difficult to conclude which of the α-helical or β-sheet structures accompanied the formation of prefibrillar aggregates by the shapes of CD spectra only, we supplementarily performed attenuated total reflection (ATR)-FTIR measurements using FT/IR-4700ST model spectrometer (Jasco, Japan) equipped with an attenuated total reference option. The measurement was performed after 120-min incubation of the 1.4 mg/ml B chain peptide solution, pH 8.7, under a quiescent condition. The B chain peptide dissolved in 10 mM NaOH was also prepared as a reference. Several microliters of the sample liquid were spotted on the ATR prism, air dried, and FTIR spectra were measured by collecting 32 interferograms with a resolution of 4 cm^{-1}at room temperature. The upper panel shows spectra at 120 min and in 10 mM NaOH, and the lower panel shows 2nd derivatives of the original spectra. Spectra were normalized so that the maximum values of amide I band ranging between 1580 and 1750 cm^{-1} were equal. The spectrum of the 120-min-incubated sample showed the presence of an absorption band at approximately 1,625 cm^{-1}, which is typical for the β-sheet structure, as well as an absorption band at approximately 1,655 cm^{-1}, indicating α-helix or random-coil structures. In contrast to this, an absorption band at approximately 1,640 cm^{-1}, which may be assigned as an α-helix structure or random-coil state, was only observed in 10 mM NaOH, which is consistent with no obvious secondary structures being confirmed on the corresponding CD spectrum (Fig. 4a). These results indicate that prefibrillar intermediates are rich in β-sheet structures.

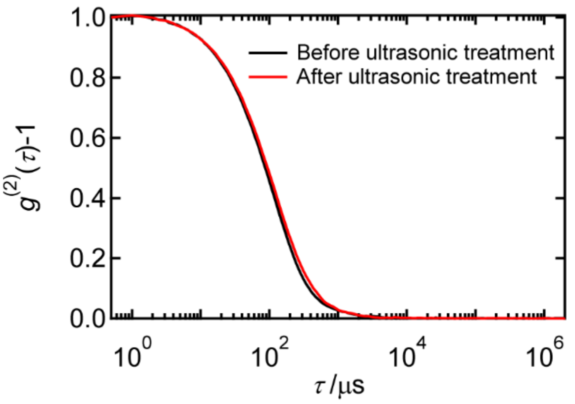

Figure S3. Intensity autocorrelation functions of DLS measurements before and after the ultrasonic treatment. The result of the ultrasonic treatment at 253 min is shown, demonstrating that no serious destruction of the particles of prefibrillar intermediates was induced under the present conditions of the ultrasonic treatment.

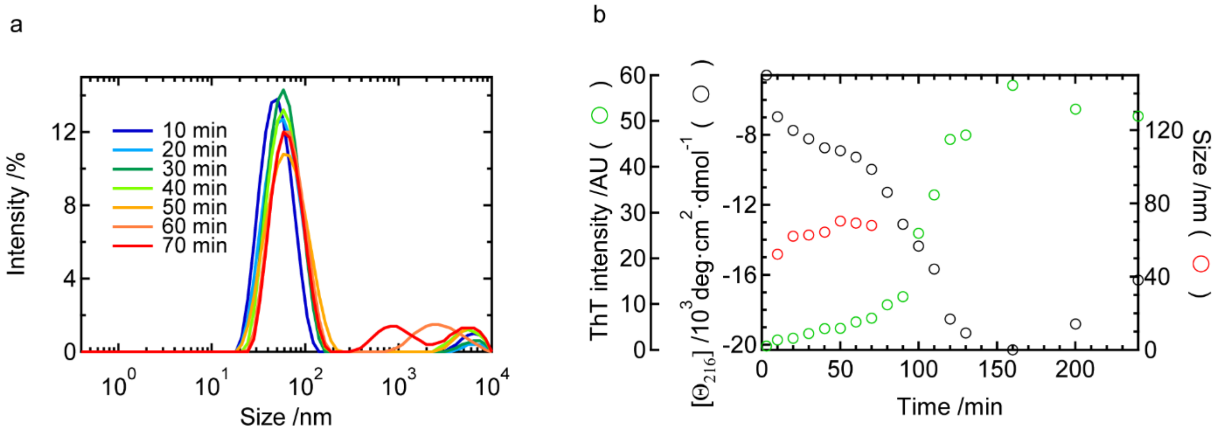

Figure S4. DLS size analysis of prefibrillar intermediates under agitated conditions. (**a**) Time-dependent changes in DLS volume-weighted size distributions in the early stage of the fibrillation reaction. Size distributions were obtained using the CONTIN method (see the Materials and Methods in the main text). Ten minutes after the start of the reaction, particles with a size of approximately 50 nm already appeared and gradually increased as a function of time. (**b**) Time evolution of the position of the main peak in the size distribution of DLS. The corresponding time courses of [θ_{216}] and ThT fluorescence intensity, which are the same as the data shown in Fig. 2d, are also shown in the figure. After ~80 min, corresponding to the time period when amyloid fibrils began to form, the tail of the intensity autocorrelation function began to stretch to a higher delay-time region and the shape then markedly shifted toward the higher delay-time region at a scale of more than 10-fold, indicating the occurrence of huge fibrils. Since the size distribution of these aggregates was difficult to calculate, we confined the analysis to the time region between 10 and 70 min. Overall, the sizes of prefibrillar intermediates appeared to be smaller than those of the 2nd prefibrillar intermediate (i.e., ~130 nm) and similar to that of the 1st intermediate (i.e., ~70 nm), suggesting that the growth of fibrils rapidly started after a small amount of the 2nd prefibrillar intermediate species was produced, and the elongation rate after the generation of amyloid nuclei may be accelerated by a combination with agitation-induced fragmentation.

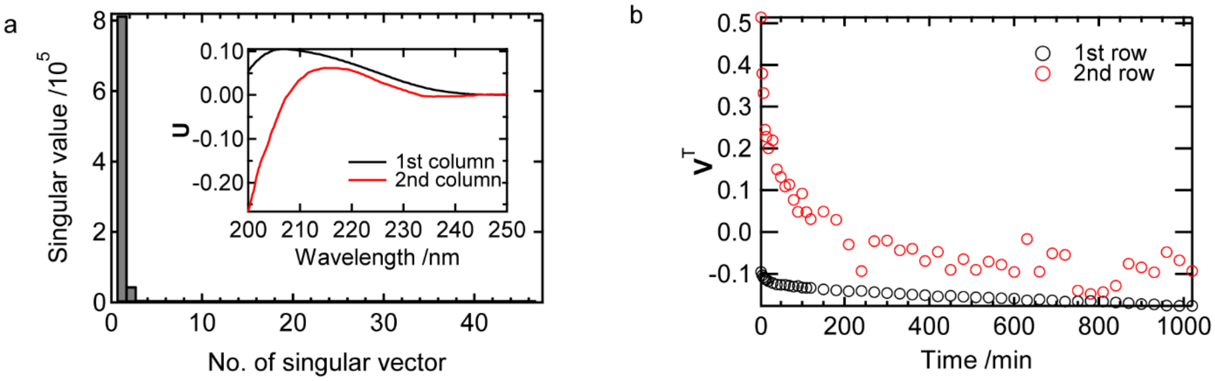

Figure S5. Singular value decomposition (SVD) analysis of CD spectra. A SVD analysis of CD spectra monitored during the fibril formation reaction under quiescent conditions, as shown in Fig. 4a, was performed to extract components that may contribute to this behavior. Regarding the brief procedure of the SVD analysis, a series of time-course CD spectra, **M**, were decomposed as follows:

$$\mathbf{M} = \mathbf{U}\mathbf{W}\mathbf{V}^{\mathrm{T}} \qquad \text{(S3)}$$

where **U**, **W**, and $\mathbf{V}^{\mathrm{T}}$ represent the left-singular matrix, singular matrix, and right-singular matrix of the data-set matrix **M**, respectively, assuming that **M** is a m × n matrix, **U** is a m × m unitary matrix, **W** is a m × n diagonal matrix, and $\mathbf{V}^{\mathrm{T}}$ is a n × n unitary matrix. In the analysis, we constructed **M** in a manner where each column was composed of wave data with the ascending delay time from the left to the right of the matrix. In this case, each column of **U** is interpreted as a normalized diagonalized wave. The weights and proportions of the waves are described by the corresponding diagonal element of **W** and corresponding row element of $\mathbf{V}^{\mathrm{T}}$, respectively.

(a) the diagonal element of the singular matrix. The inset shows the 1st and 2nd columns of the left-singular matrix. (**b**) The time course of the 1st and 2nd rows of the right-singular matrix. As shown in panel a, singular values of the 1st and 2nd components are dominant among all singular components and, thus, these two components may be sufficient to describe the spectral change. Therefore, the time dependence may be described by two independent parameters. The two left-singular vectors, which correspond to spectral components, are shown in the inset of panel a. Based on the shapes, the first spectral component was assigned to monomers with random coil structures, while the second was assigned to prefibrillar aggregates. Since the time course of the amplitudes of these components (i.e., the rows of the right-singular vectors) in panel b showed a double exponential time dependence, the second spectral component was deduced to represent the 1st and 2nd prefibrillar intermediate structures in a combined manner.

Supplementary References

1. Abelein, A.; Graslund, A.; Danielsson, J., Zinc as chaperone-mimicking agent for retardation of amyloid beta peptide fibril formation. *Proc. Natl. Acad. Sci. U. S. A.* **2015,** *112* (17), 5407-12.
2. Meisl, G.; Yang, X.; Hellstrand, E.; Frohm, B.; Kirkegaard, J. B.; Cohen, S. I.; Dobson, C. M.; Linse, S.; Knowles, T. P., Differences in nucleation behavior underlie the contrasting aggregation kinetics of the Aβ40 and Aβ42 peptides. *Proc. Natl. Acad. Sci. U. S. A.* **2014,** *111* (26), 9384-9.
3. Chatani, E.; Yagi, H.; Naiki, H.; Goto, Y., Polymorphism of β_2-microglobulin amyloid fibrils manifested by ultrasonication-enhanced fibril formation in trifluoroethanol. *J. Biol. Chem.* **2012,** *287* (27), 22827-37.
4. Hortschansky, P.; Schroeckh, V.; Christopeit, T.; Zandomeneghi, G.; Fandrich, M., The aggregation kinetics of Alzheimer's β-amyloid peptide is controlled by stochastic nucleation. *Protein Sci.* **2005,** *14* (7), 1753-9.
